# Cure for tantrums? Longitudinal associations between parental digital emotion regulation and children's self-regulatory skills

**DOI:** 10.3389/frcha.2024.1276154

**Published:** 2024-06-28

**Authors:** V. Konok, M.-A. Binet, Á. Korom, Á. Pogány, Á. Miklósi, C. Fitzpatrick

**Affiliations:** ^1^ Department of Ethology, Faculty of Science, Eötvös Loránd University, Budapest, Hungary; ^2^ Faculty of Medicine and Health Sciences, Université de Sherbrooke, Sherbrooke, QC, Canada; ^3^ Faculty of Science, Doctoral School of Biology, Eötvös Loránd University, Budapest, Hungary; ^4^ ELKH-ELTE Comparative Ethology Research Group, Budapest, Hungary; ^5^ Faculty of Education, Université de Sherbrooke, Sherbrooke, QC, Canada

**Keywords:** emotion regulation, self-regulation, digital devices, longitudinal, effortful control, impulsivity

## Abstract

**Introduction:**

Parents often use digital devices to regulate their children's negative emotions, e.g., to stop tantrums. However, this could hinder child development of self-regulatory skills. The objective of the study was to observe bidirectional longitudinal associations between parents' reliance on digital devices to regulate their child's emotions and self-regulatory tendencies (anger/frustration management, effortful control, impulsivity).

**Methods:**

Parents (*N* = 265) filled out the Child Behavior Questionnaire—Short Form and the Media Assessment Questionnaire twice: the initial assessment (T1) took place in 2020 (mean child age = 3.5 years old), and follow-up (T2) occurred a year later in 2021 (mean child age = 4.5 years old).

**Results:**

Higher occurrence of parental digital emotion regulation (PDER) in T1 predicts higher anger and lower effortful control in T2, but not impulsivity. Higher anger in T1, but not impulsivity and effortful control, predicts higher PDER in T2.

**Discussion:**

Our results suggest that parents of children with greater temperament-based anger use digital devices to regulate the child's emotions (e.g., anger). However, this strategy hinders development of self-regulatory skills, leading to poorer effortful control and anger management in the child.

## 1 Introduction

Digital device use among young children is markedly increasing. Children are introduced to screens at earlier and earlier developmental stages (1). Screen-based activities occupy the largest part of children's free time, compared to outdoor play or other screen-free activities (2). Pre-pandemic studies report that preschoolers spend about 1.5–2.5 h in front of a screen daily (3, 4), and this amount has increased by one hour during the pandemic (5). The proportion of families that possess mobile devices has increased from 52% in 2011 to 98% in 2020 and almost half of 2- to 4-years-olds have their own mobile device (6). About 26% of children aged 0–4 from the United States spend more than 4 h in front of a screen daily, meaning a two-fold increase compared to 13% before the pandemic (7). Despite these tendencies the effects of screen time on child emotional and cognitive development are still debated and largely unknown (8). The widespread usage of electronic media and digital devices (TV, videogames, PC, smartphones, tablets, etc.) may influence even adults' cognition, emotions, and mental health (9, 10). However, young children's brain and cognitive processes are still plastic, making them even more potentially vulnerable to strong and long-lasting influences of experiences (11–13).

Early childhood is a critical time for learning basic self-regulation skills (14). Self-regulation is conceptualized as the organization or modulation of affective, mental, and behavioral responses, including control over emotional experiences and expressions (i.e., emotion regulation), cognitive processes (i.e., executive function), and approach or withdrawal behaviors (i.e., effortful control) (15). Executive function and effortful control are related and, according to some researchers, overlapping constructs (16, 17). Effortful control has been defined as children's ability to inhibit a dominant response in favor of a subdominant one, or an automatic response in favor of a deliberate one (18, 19). It involves the management of attention or behavior. Effortful control is temperamentally based, but also develops with considerable input from the environment (19) especially through children's early social relationships with their parents (20, 21).

Emotion regulation involves implicit or explicit attempts to modify the natural trajectory of one or more parameters of emotion (22), including physiological arousal, expression, intensity or duration. It is related to temperamental emotional reactivity [high reactivity hinders its effectiveness (23);], and to coping [i.e., the ability to cope with the stressful situation (24, 25)]. Emotion regulation emerges in rudimentary forms in infancy (26), and gradually progresses from being a highly external process to an internal one over time (25). Certain early childhood experiences, e.g., appropriate family interactions are necessary for this developmental process (27).

In the past decades, digital devices have become increasingly prevalent in people's lives and became objects with which emotions, cognition, and behavior can be regulated. Therefore, devices and screen-based activities have become external tools of self-regulation. For example, digital activities (e.g., videogaming, watching videos, instant messaging) often serve an emotion-regulating purpose in adults [“digital emotion regulation”; (28); for a review: (29)]. They help in coping with or recovery from negative emotions and stress by providing a sense of mastery and control (29, 30), an immersive or “flow” experience (31–33), and by providing a distraction from real-life problems or escape from reality into the virtual world (34). Digital activities offer instant rewards (35, 36) which can modulate one's mood and emotions. Digital activities can also help in arousal-regulation for individuals with lower arousal by offering stimulation through e.g., fast-paced, intensive, simultaneous stimuli (8, 37) or arousing (e.g., violent) contents, which activate the dopamine and the reward pathways (8).

Parents often give digital devices to their child to “safely” engage them (“baby-sitter” function), and to regulate their emotions or behavior (38–40). Kabali et al. (41) found that 65% of parents use mobile devices to keep their child calm in public places. Television is also commonly used as a calming tool for children (42, 43). We refer to this phenomenon as parental digital emotion regulation (PDER), designating parental behaviors such as giving the child a digital device to regulate their negative emotions or calm them down.

Providing children with digital devices as “digital pacifiers” (41) may reduce child emotional expressions in the short term and may help parents allocate resources to necessary tasks and provide them with free time, especially during lockdown (44). However, this practice may also lead to missed opportunities to teach adaptive emotion regulation and coping strategies to the child (45). Although the distraction of attention away from stressful stimuli or negative emotions can be an effective short-term strategy to reduce emotional intensity in young children (46), suppressing emotions can have paradoxical, rebounding effects [e.g., (47)], and it may lead to maladaptive, avoidant coping strategies, increased negative emotionality or dysregulation in the future. Additionally, for children with immature self-regulatory skills, it may be harmful to become accustomed to external devices to regulate their emotions, as this could interfere with the development of internal regulatory mechanisms. Dependence on the device may lead to problematic media use and “screen time tantrums” (48), i.e., extreme emotions when media is removed (40). In addition, when digital devices are used for getting instant rewards (35, 36), it may hinder one's ability to delay gratification and control impulses (49, 50). This may lead to a positive feedback loop. As a consequence, an association is frequently found between media use and impulsivity (51–53) or poorer executive functions (54–57).

Empirical evidence suggests a negative association between digital media use and self-regulation. For example, more time spent watching TV was associated with higher ratings on negative emotionality, emotional reactivity, aggression, and attention problems, as well as lower levels of soothability in toddlers (58). Children who began using screen media devices earlier or who spent more time engaging with mobile devices displayed lower self-regulation (59). Longitudinal data mainly suggest bidirectional relationships. For example, screen time or digital media use at a younger age was negatively associated with later child self-regulation or related processes, such as executive function and effortful control (37, 55, 57, 60–62). However, findings also support the reverse association: emotion dysregulation and poor self-regulation was found to contribute to greater and more problematic media use later (37, 42, 63, 64). However, whether bidirectional associations exist between the use of PDER and child self-regulation remains unknown.

The few studies investigating PDER suggest its potential role in child self-regulatory skills development. Coyne et al. (40) found that temperamental dysregulation risk factors, specifically negative affectivity and surgency (65, 66), were related to problematic media use and screen time tantrums through PDER. This suggests that difficult temperament (entailing low self-regulation skills) leads to PDER, which, in turn, leads to problematic media use. In line with this, children with social-emotional difficulties, poor self-regulation and a difficult temperament have a higher chance of being given digital technology as a calming tool or as a baby-sitter (39, 42, 43, 67, 68) and perhaps as a result, they use more media later (37, 62, 64, 69).

However, as far as we know, only one longitudinal study (69) investigated the bidirectional associations between PDER and child self-regulation. This study found an interaction effect: PDER in an earlier time point (T1) was positively related to increases in children's negative emotionality in a later time point (T2), but only for children with initially low negative emotionality. Further longitudinal studies are needed as the above-mentioned results are not in line with the frequently found bidirectional association between media use and self-regulation. Therefore, the objective of the study was to observe bidirectional longitudinal associations between PDER and child self-regulatory tendencies (anger/frustration management, effortful control, impulsivity). Since self-regulatory skills are still immature in the preschool age, PDER can have a great impact on them. Therefore, we aimed to investigate this age group. We also accounted for the confounding effects of parenting stress, child screen time, and family sociodemographics.

This is a confirmatory study with clear hypotheses: we expect that poorer child self-regulatory tendencies (i.e., higher level of anger/frustration management problems, impulsivity and lower level of effortful control) (at T1) lead to higher PDER (at T2), and that higher PDER (at T1) leads to poorer child self-regulatory tendencies (higher anger/frustration management problems and impulsivity, and lower level of effortful control) (at T2).

## 2 Materials and method

### 2.1 Procedure and participants

This study is part of a larger, two-year longitudinal study on digital media use by Canadian families with preschool children aged 2–5 years during the COVID-19 pandemic. Participating families were recruited using eye-catching posters and flyers in preschools and pre-kindergarten classes, sign-up sheets and presentations given at preschool and pre-kindergarten registration nights, a Facebook page, and newspaper and radio advertisements broadcast across Nova Scotia (Canada). To measure bidirectional associations between parental digital emotion regulation and child self-regulation tendencies, we measured these variables at two time points.

**Figure 1:**
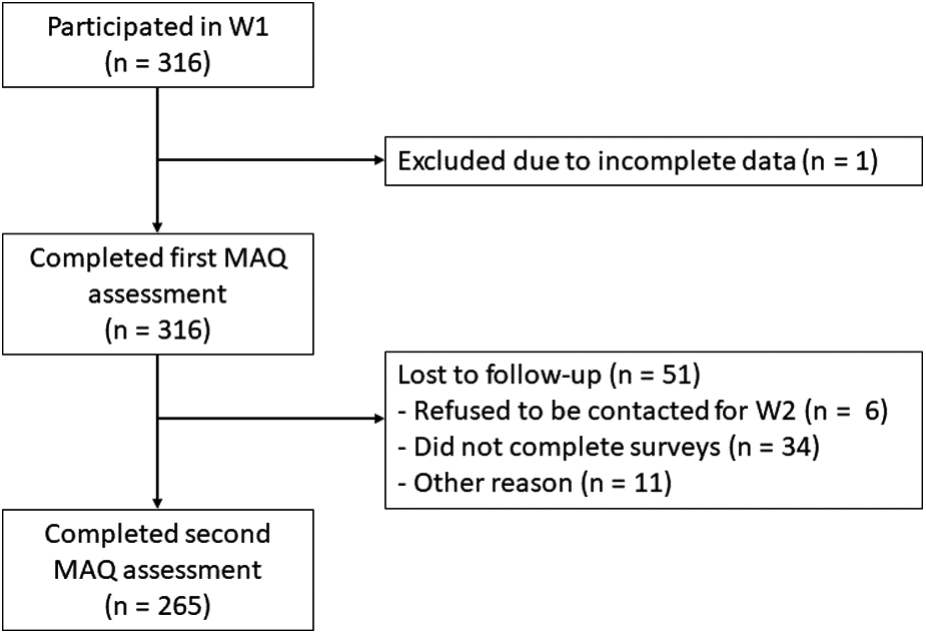
Flow diagram showing the number of participants contacted, lost for specific reasons and retained.

The first assessment took place between March and August of 2020 (*N* = 316 children), during a provincially declared state of emergency and lockdown. A follow-up with this sample was conducted a year later, between April and August of 2021 (*N* = 265; a flow diagram of the participants is presented in Figure 1). Demographics for the retested sample are presented in Table 1.

**Table 1:** Descriptive statistics of the final sample (*N* = 265).

Variable	Measure	*N* (%)	M ± SD (Min, Max)
Child age T1	In years	*N* (%) missing = 1 (0.4)	3.46 ± 0.84 (2, 5.42)
Child age T2	In years	*N* (%) missing = 0	4.33 ± 0.86 (2.75, 6.33)
Child sex	(2 values)	*N* (%) boys = 138 (52.1) *N* (%) girls = 126 (47.5) *N* (%) missing = 1 (0.4)	
Parent age T1	In years	*N* (%) missing = 0 (0)	35.24 ± 4.28 (23, 52)
Parent age T2	In years	*N* (%) missing = 0 (0)	36.24 ± 4.28 (24, 53)
Parent sex	(2 values)	*N* (%) males = 20 (7.5) *N* (%) females = 245 (92.5) *N* (%) missing = 0 (0)	
Parental education	(3 values)	*N* (%) college or secondary degree = 62 (23.4)	
		*N* (%) bachelor's degree = 129 (48.7)	
		*N* (%) master or doctoral degree = 74 (27.9) *N* (%) missing = 0 (0)	
Yearly income	(3 values)	*N* (%) $59,999 and less = 40 (15.1)	
		*N* (%) 60,000–$99,999 = 72 (27.2)	
		*N* (%) $100,000 and more = 136 (51.3) *N* (%) missing = 17 (6.4)	
Race	(7 values)	*N* (%) Aboriginal = 2 (0.8) *N* (%) Asian = 5 (1.9) *N* (%) Black = 2 (0.8) *N* (%) White = 242 (91.3) *N*% Don't know = 0 (0) *N* (%) Prefer not to answer = 1 (0.4) *N* (%) Other = 12 (4.5) *N* (%) missing = 1 (0.4)	
Country of birth	(2 values)	*N* (%) Canada (country) = 239 (90.2) *N* (%) Other = 25 (9.4) *N* (%) missing = 1 (0.4)	
Marital status	(6 values)	*N* (%) Married = 218 (82.3) *N* (%) Single/Never married = 11 (4.2) *N* (%) Live-in partner = 28 (10.6) *N* (%) Divorced = 2 (0.8) *N* (%) Widowed = 0 (0) *N* (%) Separated = 5 (1.) *N* (%) missing = 1 (0.4)	
Child screen time T1	In hours/day	*N* (%) missing = 0 (0)	3.45 ± 2.45 (0, 10.43)
Child screen time T2	In hours/day	*N* (%) missing = 0 (0)	3.26 ± 2.38 (0, 9.65)
Parenting stress T1	(total score 0–5)	*N* (%) missing = 0 (0)	1.85 ± 0.53 (1, 3.76)
Parenting stress T2	(total score 0–5)	*N* (%) missing = 0 (0)	1.14 ± 0.72 (0, 3.11)
CBQ anger T1	(total score 0–7)	*N* (%) missing = 0 (0)	4.25 ± 1.11 (1, 7)
CBQ anger T2	(total score 0–7)	*N* (%) missing = 0 (0)	4.26 ± 1.15 (1, 6.67)
CBQ impulsivity T1	(total score 0–7)	*N* (%) missing = 0 (0)	4.43 ± 0.93 (1.67, 7)
CBQ impulsivity T2	(total score 0–7)	*N* (%) missing = 0 (0)	4.19 ± 0.94 (1.33, 6.83)
CBQ effortful control T1	(total score 0–7)	*N* (%) missing = 0 (0)	4.71 ± 0.82 (2.58, 6.83)
CBQ effortful control T2	(total score 0–7)	*N* (%) missing = 0 (0)	4.88 ± 0.82 (2.75, 7)
Parental digital emotion regulation T1	(after merging: 2 values)	*N* (%) never/rarely = 165 (62.3) *N* (%) regularly/frequently = 100 (37.7) *N* (%) missing = 0 (0)	
Parental digital emotion regulation T2	(after merging: 2 values)	*N* (%) never/rarely = 179 (67.5) *N* (%) regularly/frequently = 86 (32.5) *N* (%) missing = 0 (0)	

At both time waves, parents completed the web-based Media Assessment Questionnaire (MAQ), which has been described in detail elsewhere (70). The MAQ assesses child and parent media use and includes questions on child age and sex, parent education, as well as reasons reported by parents to allow their child to use media. For the purpose of this study, we integrated items on child temperamental anger/frustration, impulsivity and effortful control using the Child Behavior Questionnaire—Short Form (71). We also integrated items on parenting stress using the Parenting Stress Index (72). These measures are described below. The use of data for this specific study received approval from the ethics board from the principal investigator's institution (IRB #2021-2927). Informed consent to participate was obtained from participating parents.

### 2.2 Measures

#### 2.2.1 Parental digital emotion regulation (PDER)

Parents were asked to rate how much they agreed or disagreed with the statement “I let my child use media to calm them down when they are upset”. Responses were rated on a 7-point Likert scale ranging from *Never* (1) to *Several times per day* (7). Due to some of the response options having very small frequencies (e.g., “Several times per day”: *N* = 4), this variable was then recoded into a dichotomous variable (1 = Never/rarely, 2 = Regularly/frequently; see Table 1 for descriptives).

#### 2.2.2 Child self-regulatory tendencies

The Child Behavior Questionnaire—Short form (71) assesses several distinct dimensions of temperament which are grouped into three main factors: negative affectivity, surgency/extraversion, and effortful control. The short form shows satisfactory internal consistency, criterion validity, longitudinal stability and inter-rater agreement (71, 73). Since we focus on temperament-based self-regulatory tendencies, we retained for this study (1) anger/frustration (“anger” hereinafter) which is a dimension belonging to the negative affectivity main factor, (2) impulsivity which is a dimension belonging to the surgency/extraversion main factor, and finally (3) the main factor of effortful control. Anger (e.g., “Child gets angry when told s/he has to go to bed”) and impulsivity (e.g., “Usually rushes into an activity without thinking about it”) were each based on the mean of 6 items. Higher scores in anger and impulsivity subscales indicate greater intensity and duration of the child's angry or frustrated response to environmental stimuli or greater speed of response initiation, respectively. The effortful control factor was based on mean scores obtained for the dimensions of attentional focusing (six items, e.g., “When drawing or coloring a book, shows strong concentration”) and inhibitory control (six items, e.g., “Can wait before entering into new activities if s/he is asked to”). Higher scores in the attentional focusing and the inhibitory control subscales indicate better child effortful control. Items are scored on a 7-point Likert scale ranging from 1 (extremely untrue of your child) to 7 (extremely true of your child). The Cronbach's alpha coefficients for anger, impulsivity, and effortful control in Wave 1 were *α* = 0.789; 0.629 and 0.792, respectively. In Wave 2, the corresponding coefficients were *α* = 0.814; 0.656 and 0.785, respectively (see Table 1 for descriptives).

#### 2.2.3 Demographics, parenting stress and child screen time

When completing the MAQ (70), parents reported child age and sex (assigned at birth), parent age and sex, parent education, yearly income, race, country of birth, marital status, and parenting stress. Race, country of birth and marital status were not used in the analyses because there was little variance on these variables (see Table 1 for response options and number/percentage of participants answering them). Parenting stress was assessed using the Parenting Stress Index (72). This questionnaire includes a Parental distress (PD) subscale (12 items, i.e., “I find myself giving up more of my life to meet my child's needs than I ever expected”) and a Parent-child dysfunctional interaction (PCDI) subscale (12 items, i.e., “My child smiles at me much less than I expected”). Items were rated on a 5-point Likert scale as: 1 (strongly disagree); 2 (disagree); 3 (not sure); 4 (agree) or 5 (strongly agree) and were then averaged to create a total score ranging from 1 to 5, with an adequate internal consistency (Cronbach's alpha = 0.850). Higher scores indicate higher parenting stress (see Table 1 for descriptives).

In the MAQ, parents also reported their child screen time by reporting the average amount of time their child spent doing each of the following activities: (1) Watching TV or DVDs; (2) Using a computer; (3) Playing video games on a console; (4); Using an iPad, tablet, LeapPad, iTouch, or similar mobile device (excluding smartphones); or (5) Using a smartphone. For each activity, response options were: (1) Never; (2) Less than 30 min; (3) 30 min to 1 h; (4) 1–2 h; (5) 2–3 h; (6) 3–4 h; (7) 4–5 h; and (7) more than 5 h. Parents reported this separately for a typical weekday and a typical weekend day. Total amount of child screen time was calculated by summing the durations for each activity, using responses' mid-points with the exception of “Never” and “more than 5 h” where scores of 0 and 5 were used, respectively. To compute child average daily screen time, we computed a weighted average of screen time across the week as follows: [(weekday screen time X 5) + (weekend screen time X 2)]/7 (see Table 1 for descriptives).

### 2.3 Data analytic strategy

First, we compared whether retained (those who participated at T2) and unretained (those who had dropped out) participants were different in any aspects of demographics, parental digital emotion regulation (PDER), or child self-regulation scores (Mann–Whitney tests, *t*-tests and *χ*^2^ tests, using SPSS 28.0.0.0).

Of the final retained sample of 265, 17 participants had a missing value on income and one participant had a missing value for child anger, impulsivity, and effortful control at T2. We performed Little's test to determine if data were missing completely at random (MCAR). The test was not significant revealing that data could be assumed to be MCAR: *χ*^2 ^= 24.476, DF = 26, *p* = 0.549.

We imputed missing values on income to the median (value of 3) and used FIML (maximum likelihood information) to account for missing outcome data.

**Figure 2:**
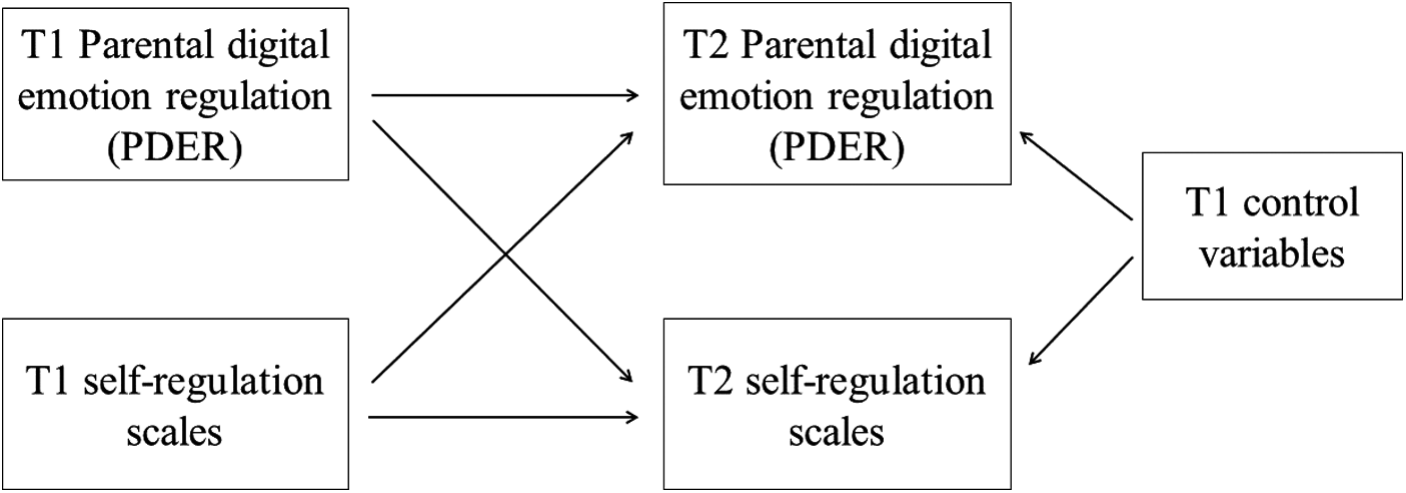
Summary of the cross-lagged panel analyses.

To test bidirectional associations between PDER and self-regulation scales, we estimated cross-lagged panel models using Mplus version 8.10 (74). We controlled for sociodemographic variables such as child age and sex, parent age and sex, parent education, yearly income, child screen time and parenting stress (the model schema is presented on Figure 2).

## 3 Results

### 3.1 Comparing retained and unretained participants

Unretained participants were significantly different from retained participants in parents' age (M ± SD = 33.32 ± 4.631 (unretained) vs. 35.226 ± 4.278 years (retained); *t* = 2.852; *p* = 0.005; Cohen's *d* = 0.436) and marginally in PDER (52% vs. 37.6% of regular/frequent PDER in unretained and retained sample, respectively; *χ*^2 ^= 3.643; *p* = 0.056; this is discussed in the limitation section), but was not significantly different on any other demographic variables or child behavior variables (all *p* > 0.170).

### 3.2 Cross-lagged panel model: anger

**Figure 3:**
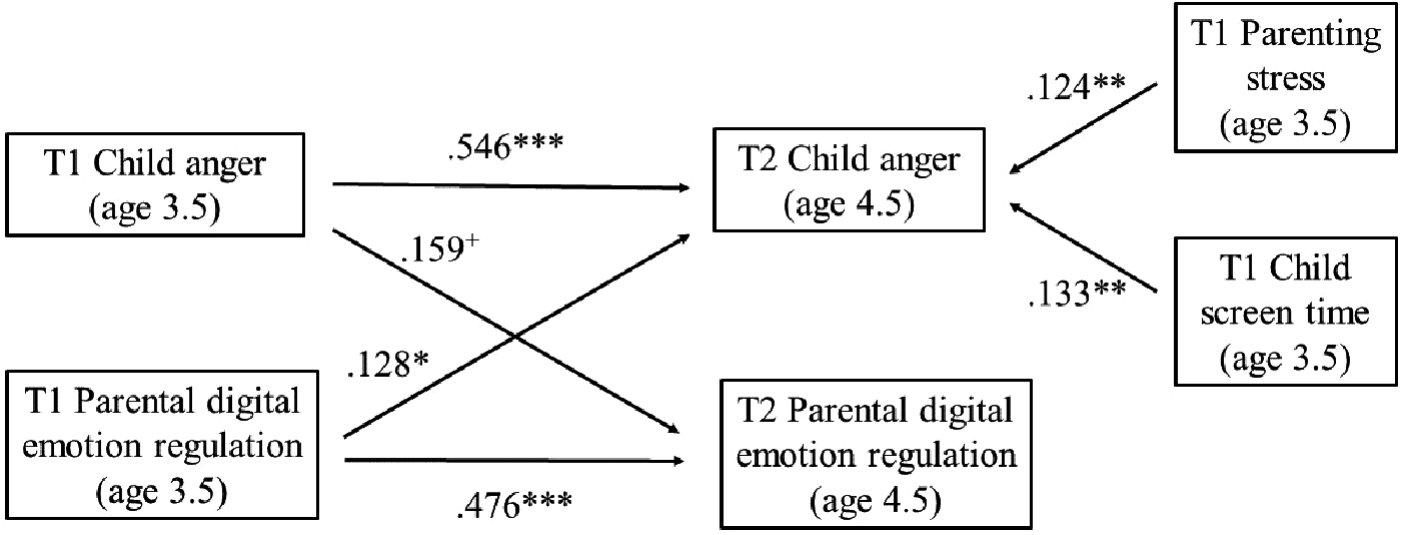
Longitudinal cross-lagged associations between parental digital emotion regulation and the anger/frustration dimension of the child behavior questionnaire.

Our final model is presented in Figure 3. Our cross-lagged panel model provided good fit [CFI = 1.000; TLI = 1.000; RMSEA = 0.000 (0.000; 0.138)] and accounted for 36.7% and 45.8% of the variance in PDER and Anger at T2, respectively. Analyses revealed considerable stability in PDER (*b* = 1.233; SE = 0.187; *p* < 0.001; *β* = 0.476) and Anger (*b* = 0.567; SE = 0.054; *p* < 0.001; *β* = 0.546) between T1 and T2. In terms of the cross-lagged associations, T1 PDER significantly contributed to higher Anger at T2 (*b* = 0.304; SE = 0.122; *p* = 0.013; *β* = 0.128), whereas T1 Anger only tendentiously contributed to higher PDER at T2 (*b* = 0.180; SE = 0.108; *p* = 0.094; *β* = 0.159). Parenting stress (*b* = 0.018; SE = 0.007; *p* = 0.008; *β* = 0.124) and child screen time (*b* = 0.063; SE = 0.024; *p* = 0.009; *β* = 0.133) at T1 were also significantly positively associated with Anger at T2 (Table 2).

**Table 2:** Results of the cross-lagged panel model measuring the bi-directional associations between PDER (parental digital emotion regulation) and the anger/frustration dimension of the child behavior questionnaire.

	Estimate (b)	se	*p*-value	Beta
Child age → T2 PDER	−0.168	0.118	0.155	−0.112
Child sex → T2 PDER	−0.064	0.190	0.737	−0.025
T1 screen time → T2 PDER	0.054	0.043	0.209	0.105
Parent age → T2 PDER	−0.022	0.025	0.359	−0.077
Parent sex → T2 PDER	0.018	0.382	0.962	0.004
Parent education → T2 PDER	0.127	0.146	0.384	0.073
Yearly income → T2 PDER	−0.048	0.124	0.700	−0.028
T1 parenting stress → T2 PDER	−0.002	0.013	0.870	−0.013
T1 PDER → T2 PDER	1.233	0.187	0.000	0.476
T1 Anger → T2 PDER	0.180	0.108	0.094	0.159
Child age → T2 Anger	−0.021	0.072	0.768	−0.015
Child sex → T2 Anger	−0.066	0.113	0.558	−0.029
T1 screen time → T2 Anger	0.063	0.024	0.009	0.133
Parent age → T2 Anger	0.020	0.014	0.153	0.073
Parent sex → T2 Anger	−0.314	0.178	0.078	−0.072
Parent education → T2 Anger	0.066	0.082	0.425	0.041
Yearly income → T2 Anger	0.036	0.081	0.658	0.023
T1 parenting stress → T2 Anger	0.018	0.007	0.008	0.124
T1 anger → T2 Anger	0.567	0.054	0.000	0.546
T1 PDER → T2 Anger	0.304	0.122	0.013	0.128

### 3.3 Cross-lagged panel model: impulsivity

**Figure 4:**
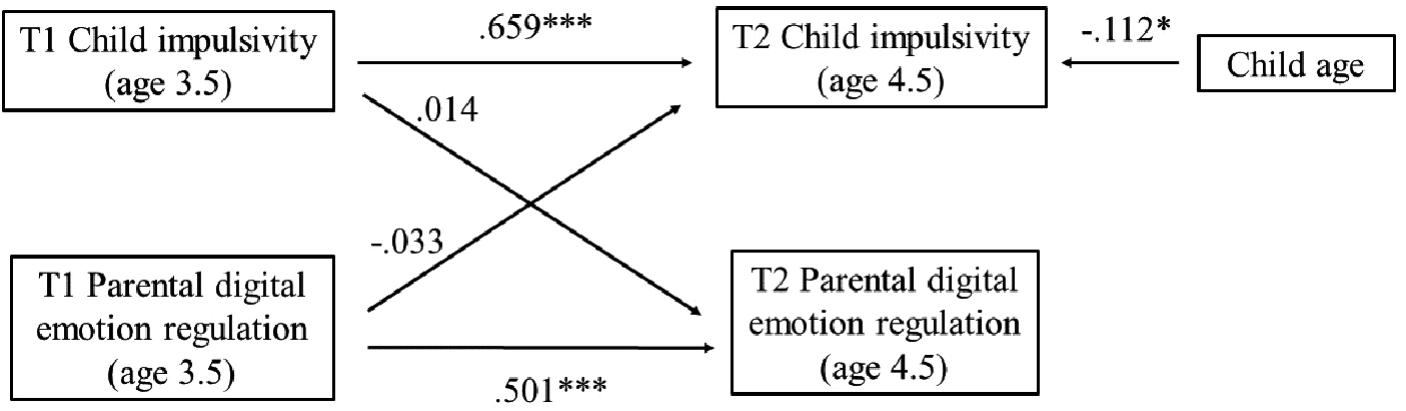
Longitudinal cross-lagged associations between parental digital emotion regulation and the impulsivity dimension of the child behavior questionnaire.

Our model is presented in Figure 4. Our cross-lagged panel model provided good fit [CFI = 1.000; TLI = 1.000; RMSEA = 0.000 (0.000; 0.078)] and accounted for 34.7% and 47.8% of the variance in PDER and Impulsivity at T2, respectively. Analyses revealed considerable stability in Impulsivity (*b* = 0.670; SE = 0.051; *p* < 0.001; *β* = 0.659) between T1 and T2. In terms of the cross-lagged associations, neither T1 PDER was associated with T2 Impulsivity (*b* = −0.064; SE = 0.094; *p* = 0.496; *β* = −0.033), nor T1 Impulsivity with T2 PDER (*b* = 0.019; SE = 0.096; *p* = 0.845; *β* = 0.014). Child age was significantly negatively associated with Impulsivity at T2 (*b* = −0.126; SE = 0.057; *p* = 0.028; *β* = −0.112; Table 3).

**Table 3:** Results of the cross-lagged panel model measuring the bi-directional associations between PDER (parental digital emotion regulation) and the impulsivity dimension of the child behavior questionnaire.

	Estimate (b)	se	*p*-value	Beta
Child age → T2 PDER	−0.167	0.118	0.158	−0.114
Child sex → T2 PDER	−0.059	0.189	0.755	−0.024
T1 Screen time → T2 PDER	0.064	0.043	0.138	0.127
Parent age → T2 PDER	−0.025	0.025	0.304	−0.087
Parent sex → T2 PDER	−0.007	0.395	0.986	−0.001
Parent education → T2 PDER	0.117	0.145	0.422	0.068
Yearly income → T2 PDER	−0.037	0.121	0.761	−0.022
T1 parenting stress → T2 PDER	0.006	0.012	0.597	0.039
T1 PDER → T2 PDER	1.276	0.185	0.000	0.501
T1 Impulsivity → T2 PDER	0.019	0.096	0.845	0.014
Child age → T2 Impulsivity	−0.126	0.057	0.028	−0.112
Child sex → T2 Impulsivity	−0.122	0.087	0.160	−0.065
T1 screen time → T2 Impulsivity	0.022	0.021	0.297	0.057
Parent age → T2 Impulsivity	−0.004	0.012	0.761	−0.016
Parent sex → T2 Impulsivity	0.203	0.145	0.164	0.057
Parent education → T2 Impulsivity	0.103	0.064	0.106	0.079
Yearly income → T2 Impulsivity	−0.083	0.066	0.207	−0.065
T1 parenting stress → T2 Impulsivity	−0.004	0.005	0.491	−0.031
T1 Impulsivity → T2 Impulsivity	0.670	0.051	0.000	0.659
T1 PDER → T2 Impulsivity	−0.064	0.094	0.496	−0.033

### 3.4 Cross-lagged panel model: effortful control

**Figure 5:**
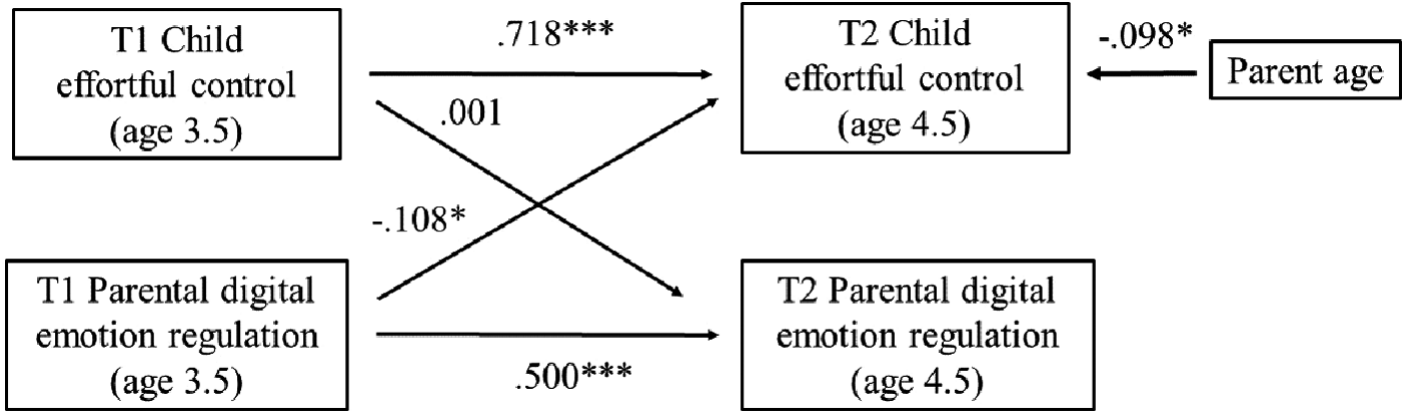
Longitudinal cross-lagged associations between parental digital emotion regulation and the effortful control main factor of the child behavior questionnaire.

Our model is presented in Figure 5. Our cross-lagged panel model provided good fit [CFI = 1.000; TLI = 1.000; RMSEA = 0.000 (0.000; 0.000)] and accounted for 34.7% and 55.5% of the variance in PDER and Effortful control at T2, respectively. Analyses revealed considerable stability in Effortful control between T1 and T2 (*b* = 0.716; SE = 0.050; *p* < 0.001; *β* = 0.718). In terms of the cross-lagged associations, T1 PDER significantly contributed to lower Effortful control at T2 (*b* = −0.182; SE = 0.075; *p* = 0.016; *β* = −0.108), whereas T1 Effortful control did not contribute to higher PDER at T2 (*b* = −0.002; SE = 0.129; *p* = 0.986; *β* = −0.001). Parent age was also significantly negatively associated with Effortful control at T2 (*b* = −0.019; SE = 0.008; *p* = 0.022; *β* = −0.098; Table 4).

**Table 4:** Results of the cross-lagged panel model measuring the bi-directional associations between PDER (parental digital emotion regulation) and the effortful control main factor of the child behavior questionnaire.

	Estimate (b)	se	*p*-value	Beta
Child age → T2 PDER	−0.168	0.120	0.160	−0.115
Child sex → T2 PDER	−0.061	0.189	0.749	−0.024
T1 Screen time → T2 PDER	0.066	0.042	0.118	0.130
Parent age → T2 PDER	−0.025	0.025	0.303	−0.088
Parent sex → T2 PDER	−0.008	0.395	0.983	−0.002
Parent education → T2 PDER	0.117	0.146	0.423	0.068
Yearly income → T2 PDER	−0.039	0.120	0.749	−0.023
T1 parenting stress → T2 PDER	0.006	0.012	0.618	0.038
T1 PDER → T2 PDER	1.275	0.186	0.000	0.500
T1 effortful control → T2 PDER	−0.002	0.129	0.986	−0.001
Child age → T2 Effortful control	−0.066	0.048	0.164	−0.068
Child sex → T2 Effortful control	0.091	0.071	0.202	0.055
T1 screen time → T2 Effortful control	−0.015	0.016	0.349	−0.044
Parent age → T2 Effortful control	−0.019	0.008	0.022	−0.098
Parent sex → T2 Effortful control	−0.108	0.139	0.437	−0.035
Parent education → T2 Effortful control	0.013	0.049	0.788	0.012
Yearly income → T2 Effortful control	0.039	0.048	0.418	0.035
T1 parenting stress → T2 Effortful control	0.005	0.006	0.383	0.046
T1 effortful control → T2 Effortful control	0.716	0.050	0.000	0.718
T1 PDER → T2 Effortful control	−0.182	0.075	0.016	−0.108

## 4 Discussion

We investigated the relationships between parental digital emotion regulation and self-regulation in children. Our study revealed complex, bidirectional longitudinal associations between the investigated variables. The results suggest that parental digital emotion regulation may contribute to the bidirectional association between media use and self-regulation in children (37, 55, 62, 63). The observed associations were consistent and strong in one direction (higher frequency of parental digital emotion regulation leading to higher anger/frustration and lower effortful control), but less consistent and more tendentious in the other direction (effortful control does not, while anger/frustration tendentiously contribute to higher PDER).

### 4.1 Higher PDER leads to poorer anger/frustration management and effortful control

Higher baseline occurrence of parental digital emotion regulation (PDER) and higher baseline screen time predicted poorer anger/frustration management (i.e., higher anger) one year later. This is in line with findings of a cross-sectional study (hence limitations regarding causal inferences) that more time spent watching TV is associated with higher levels of negative emotionality, emotional reactivity, and aggression, as well as lower levels of soothability in toddlers (58). Longitudinal studies (57, 62) found that baseline digital media use predicted more externalizing problems (specifically, conduct problems and hyperactivity) at follow-up, and these problems often entail difficulties with anger management (75–78). While these associations or effects can be driven by several mechanisms [e.g., direct effects, like overstimulation, and indirect effects, like displacement of social interactions (79)], the present study suggests that using digital devices for emotion regulation might be a key determinant in the development of child difficulties with various aspects of self-regulation. Our results somewhat contradict those of Gordon-Hacker & Gueron-Sela (69), who found in a path analysis that early maternal digital emotion regulation preceded later negative emotionality only in children with low initial negative emotionality. However, it should be noted that the authors found a significant, although weak (*r* = 0.2) longitudinal correlation between T1 maternal digital emotion regulation and T2 negative emotionality, and a slightly stronger cross-sectional correlation (*r* = 0.37) between T2 maternal digital emotion regulation and T2 negative emotionality, but neither of them were significant in the path analysis. One possible explanation for the divergent findings in their study and ours, is the different age groups of the children. Additional explanation for these somewhat contradictory results should be revealed through further longitudinal studies. Furthermore, screen time and PDER are closely related, and as the present design does not allow for the separation of the two phenomena, further research is needed to disentangle their respective effects on child self-regulation.

Higher occurrence of PDER at T1 also predicted lower levels of effortful control at T2. In line with this, longitudinal studies have found that those who spend more time using digital devices subsequently develop more attentional problems, impulsivity, and poorer executive functions or self-regulation in general (37, 55, 61, 64, 80). These results corroborate the involvement of PDER in developing self-regulation problems. Contrary to our expectations, however, PDER in T1 did not predict impulsivity in T2. This contrasts with the findings of several studies which showed that digital device use leads to hyperactivity, inattention or externalizing behaviors (81). It was also unexpected that T1 PDER predicted only effortful control, whereas impulsivity and effortful control are related constructs (16, 17). The scale of effortful control is made up of items on attentional focusing (e.g., Tendency to maintain attentional focus upon task-related channels) and inhibitory control (e.g., The capacity to plan and to suppress inappropriate approach responses under instructions or in novel or uncertain situations). On the other hand, impulsivity is defined as the “speed of response initiation” (71), consisting of items like “Usually rushes into an activity without thinking about it”. While high impulsivity entails low inhibitory control, and both are related to behavioral self-regulation, attentional focusing is a different, more cognitive construct and does not necessarily correlate with the other two (82, 83). It is possible that PDER affects attentional processes inherent to effortful control to a larger extent than behavioral self-regulation. Higher PDER is associated with higher screen time (38) and the latter may have negative effects on attentional focusing (37, 80), for example, as a result of overstimulation (84). The associations between early digital media use and later attentional problems are well supported by empirical data (37, 80), while relationships between digital media use and executive functions are more contradictory (54, 56, 61, 85–87). Therefore, further studies should investigate the longitudinal associations of PDER with attentional focusing and inhibitory control separately.

### 4.2 Poorer anger/frustration management skills in T1, but not impulsivity and effortful control, predicts tendentiously higher occurrence of parental digital emotion regulation in T2

Poorer baseline anger/frustration management skills (i.e., higher anger) tendentiously predicted higher occurrence of PDER at follow-up. This result is in line with cross-sectional findings showing that children with social-emotional difficulties, poor self-regulation and a more difficult temperament have a higher chance of being given digital technology as a calming tool or as a baby-sitter (39, 43, 67, 68) and with longitudinal studies showing that these problems lead to using more media later (37, 42, 62, 64). Our study is the first longitudinal study in support of poor emotion regulation leading to higher chances of parental digital emotion regulation, although the association was only marginally significant (*p* = 0.094). Parents with difficult children may struggle more with decreasing the tempers or negative emotions of the child. Therefore, they may turn to digital devices to alleviate their burden. As Radesky et al. (39) pointed out, “frustration with the child's behavior would lead to use of digital media as a coping strategy” (p. 397). Similarly, instrumental use of media (using media as a behavior modifier or as a babysitter) was primarily endorsed by parents who are less confident about their parenting (68), and children with difficult temperament may contribute to parents being less confident about their parenting skills (88, 89).

Impulsivity and effortful control did not predict later PDER. This suggests that parents use digital media as a parenting tool only for managing emotional self-regulation problems in the child, but not cognitive or behavioral self-regulation problems. This result is unexpected, but may reflect the fact that impulsivity and lower effortful control in the child may be less challenging for the parent than anger management problems, as the latter entails emotional outburst and tantrums. Some studies (67, 68) indicate that impulsivity and lower effortful control (specifically, conduct problems and energetic temperaments) are associated with PDER. However these studies are cross-sectional and cannot inform causal nor directional inferences. The present longitudinal study suggests that these self-regulatory tendencies do not lead to PDER, but rather the other way around as we found that PDER led to lower effortful control. Although many longitudinal studies found that children with attentional problems, higher impulsivity and lower self-regulation at baseline consume more digital media later (37, 62, 64, 79), these effects may not be driven by parental motivation to regulate the child's behavior/emotion by digital devices. Based on our results, we argue it is likely that children with these problems are more prone to use digital devices, independently of how much their parents try to regulate their behavior with the device.

### 4.3 Limitations

To draw appropriate conclusions from the results, some limitations should be addressed.

PDER was solely measured by parent report and with only one item. More elaborate measures are required in future studies to corroborate the present findings, and parent reported PDER should be validated by behavioral observations. Parents also reported child self-regulation tendencies, which could lead to shared measurement bias. Replications with reports from preschool teachers or using different methodologies engaging parents more actively to support their recall memories or opinions about their child's behavior could advance future studies.

The internal consistency for impulsivity was lower than desirable (Cronbach's alpha was 0.629 in Wave 1 and 0.656 in Wave 2). This might have reduced the statistical power of the analysis to detect associations with PDER. Additionally, the dimensions of impulsivity and anger have rarely been used separately (90). Although their reliability has been frequently proven to be satisfactory (71, 73), the validity of these subscales is less known with few existing studies showing moderate correlations with other questionnaire scales (91), and low to moderate correlations with laboratory observational measures (92, 93). In the future, more studies are required to better corroborate the construct validity of these subscales.

Another potential confounder of the results is that data collection took place during a provincially declared state of emergency and lockdown because of the Covid-19 pandemic. Since digital device use increased during lockdowns (94, 95), our findings should be replicated in post pandemic contexts.

Additionally, convenience sampling may not be representative of the general population. This decreases the generalizability of the results to the whole population. Replications with larger sample are warranted. On the other hand, random sampling makes it impossible to separate the effects of screen time and PDER (as they are closely related). Therefore, further research is needed to disentangle their effects on self-regulation.

Lastly, the unretained sample differed from the retained sample in that parents in the unretained sample were younger and marginally used more PDER than the retained participants. This might have caused a systematic bias in the sample. Those who participated in the second data collection wave might be more conscious parents (applying less PDER), and this may distort the observed associations. For example, it is possible that the association between T1 effortful control and impulsivity and T2 PDER was not significant (and between T1 anger and T2 PDER was only marginally significant) because conscious parents try to find other ways besides digital media to regulate or engage the child. These shortcomings should be addressed in future studies.

### 4.4 Conclusion

Our study is the first longitudinal study revealing bidirectional associations between parental digital emotion regulation and child emotion-regulation skills. Results support that higher anger/frustration in the child renders parents tendentiously more likely use digital devices to regulate child emotions. However, while digital emotion regulation can be effective in the short term, this strategy may hinder child development of self-regulatory skills in the long term, leading to poorer effortful control and anger management. This process may lead to a positive feedback loop, resulting in increased dependence on the digital device and potential later problematic media use, “screen time tantrums” (40), and technological addiction (96). Based on these results, efforts should be made to call parents' attention on the harmful consequences of digital emotion regulation. Pediatricians, child psychologists, health professionals, and social workers working directly with families or performing home visits should ask parents about the use of digital media in the family. Additionally, they should be especially attentive to parents of children with difficult temperament, as they may be at higher risk of using PDER. These parents should receive as much support as possible to reinforce emotion regulation methods other then PDER. In addition, peoples' awareness should be increased about digital devices being inappropriate tools for curing tantrums.

## Data Availability

The raw data supporting the conclusions of this article will be made available by the authors, without undue reservation.
